# Modular Enzymatic Cascade Synthesis of Nucleotides Using a (d)ATP Regeneration System

**DOI:** 10.3389/fbioe.2020.00854

**Published:** 2020-08-06

**Authors:** Maryke Fehlau, Felix Kaspar, Katja F. Hellendahl, Julia Schollmeyer, Peter Neubauer, Anke Wagner

**Affiliations:** ^1^Chair of Bioprocess Engineering, Institute of Biotechnology, Faculty III Process Sciences, Technische Universität Berlin, Berlin, Germany; ^2^BioNukleo GmbH, Berlin, Germany

**Keywords:** enzymatic cascade synthesis, nucleoside-5′-triphosphate, one-pot multi-enzyme reaction, nucleotide analog, nucleotide kinase, nucleoside kinase, modular, ATP regeneration system

## Abstract

Nucleoside-5’-triphosphates (NTPs) and their analogs are building blocks of DNA and are important compounds in both pharmaceutical and molecular biology applications. Currently, commercially available base or sugar modified NTPs are mainly synthesized chemically. Since the chemical production of NTPs is time-consuming and generally inefficient, alternative approaches are under development. Here we present a simple, efficient and generalizable enzymatic synthesis method for the conversion of nucleosides to NTPs. Our one-pot method is modular, applicable to a wide range of natural and modified nucleotide products and accesses NTPs directly from cheap nucleoside precursors. Nucleoside kinases, nucleoside monophosphate (NMP) kinases and a nucleoside diphosphate (NDP) kinase were applied as biocatalysts. Enzymes with different substrate specificities were combined to produce derivatives of adenosine and cytidine triphosphate with conversions of 4 to 26%. The implementation of a (deoxy)ATP recycling system resulted in a significant increase in the conversion to all NTP products, furnishing 4 different NTPs in quantitative conversion. Natural (deoxy)NTPs were synthesized with 60 to >99% conversion and sugar- and base-modified NTPs were produced with 69 to >99% and 27 to 75% conversion, respectively. The presented method is suitable for the efficient synthesis of a wide range of natural and modified NTPs in a sustainable one-pot process.

## Introduction

Modified nucleotides are important small molecules in molecular biology and pharmaceutical applications. Natural and modified nucleoside-5’-triphosphates (NTPs) are valuable building blocks for PCR, fluorescent *in situ* hybridization (FISH), aptamer production as well as for next generation sequencing (Prober et al., 1987; Zaccolo et al., 1996; Giller et al., 2003; Ni et al., 2017). Nucleotides with an azide or alkyne function enable the post-synthetic modification of oligonucleotides via click-chemistry (Gramlich et al., 2008).

Furthermore, the application of nucleotide prodrugs is of increasing interest (Pradere et al., 2014) as most of the known nucleoside analog drugs are only active as the respective nucleoside diphosphate (NDP) or NTP and activation *in vivo* is often insufficient (Deville-Bonne et al., 2010). In a number of approaches including the application of sofosbuvir or remdesivir, protected nucleoside monophosphates (NMPs) were administered to overcome the first activation step *in vivo* (Pradere et al., 2014; Ko et al., 2020). Additionally, methods have been developed for the production of NDP or NTP prodrugs with an increased biological availability of the respective nucleoside drug as shown for sofosbuvir or remdesivir (Pradere et al., 2014).

To date, nucleotides are primarily prepared by chemical methods such as the Yoshikawa protocol or the Ludwig-Eckstein method (Burgess and Cook, 2000). A common disadvantage of these multistep synthesis reactions is a limited regioselectivity leading to the formation of different phosphorylation products, as well as the limited control over the exclusive formation of the triphosphate, as higher phosphorylation products can be generated. The overall process is laborious, involves toxic reagents and often achieves only moderate product yields. Furthermore, some nucleotide products harboring sensitive functional groups are not accessible in these approaches as they do not withstand the harsh reaction conditions and generate undesired side products (Lee and Momparler, 1976; Burgess and Cook, 2000).

Biocatalytic routes exploiting enzymes from the nucleotide metabolism promise improved regioselectivity and exquisite control over the phosphorylation product. Driven by these advantages, several attempts have been made to replace chemical synthesis routes for NTP preparation. Natural (deoxy)NTPs have been produced by isolation from animal extracts (Berger, 1956), phosphorylation of RNA/DNA degradation products (Haynie and Whitesides, 1990) or by enzymatic cascade reactions (Ding et al., 2019). While the described methods are suitable for the synthesis of natural NTPs they cannot be easily transferred to the synthesis of NTP analogs bearing sugar or base modifications. Therefore, specific enzymatic synthesis routes were only rarely developed for few NTP analogs (Lee and Momparler, 1976; Da Costa et al., 2007; Hennig et al., 2007).

The application of nucleosides as substrates for the synthesis of nucleotides together with purified biocatalysts offers significant advantages over the approaches described above. Natural nucleosides are generally cheaply available as commercial reagents and modified nucleosides can be accessed enzymatically via known methods in high yield (Kamel et al., 2019; Kaspar et al., 2020; Yehia et al., 2020). The use of purified enzymes minimizes side reactions and simplifies reaction workup. Despite these advantages, however, there are only few examples in the literature of NTP synthesis from nucleosides with purified enzymes. As an example Baughn et al. (1978) used purified adenosine kinase, adenosine monophosphate kinase, acetate kinase and acetyl phosphate to convert adenosine to adenosine triphosphate (ATP) in a one-pot approach with 80% conversion. However, small amounts of nucleotide products ATP, adenosine diphosphate (ADP) and adenosine monophosphate (AMP) were needed to start the reaction. To our knowledge, only a single one-pot approach for the synthesis of a modified NTP from a nucleoside has been described by Hennig et al. (2007) who produced 5-fluorocytidine triphosphate (5F-CTP, **4c**). The compound was prepared with 78% isolated yield using a uridine kinase, nucleoside monophosphate kinase, pyruvate kinase as well as an enolase and 3-phosphoglycerate mutase. ATP and 3-phosphoglycerate were used as phosphate donors.

Phosphate donor recycling systems promise increased yields in these reactions by shifting the reaction equilibrium toward the desired product NTP (Abu and Woodley, 2015). These recycling systems are coupled enzymatic reactions which constantly (re)generate NTPs from the respective NDPs and a secondary phosphate donor. Indeed, the incorporation in nucleotide synthesis reactions has been described to enable higher conversions via an equilibrium shift (Wu et al., 2003). The recovery of (d)ATP from (d)ADP lowers substrate and product inhibition effects by decreasing the concentration of the side product (d)ADP. At the same time, the concentration of (d)ATP is kept high to facilitate the kinase reactions. Further advantages of phosphate donor recycling systems include simplified product purification and cost savings by decreasing the amount of (d)ATP required for high conversions.

For the enzymatic regeneration of (d)NTPs acetate kinase, pyruvate kinase and polyphosphate kinase are most commonly used (Endo and Koizumi, 2001; Andexer and Richter, 2015). Those enzymes possess high substrate promiscuity and are able to (re)generate most natural and some modified (d)NTPs from the respective (d)NDPs (Ishige et al., 2001; Gao et al., 2008; Zou et al., 2013). For example, pyruvate kinase has been applied for both the conversion of 5-fluorinated NDPs to 5-fluorinated NTPs as well as the regeneration of the phosphate donor ATP for other coupled enzymatic reactions (Hennig et al., 2007). Regeneration systems also offer the opportunity to reduce the concentration of non-ATP phosphate donors as shown for the enzymatic synthesis of natural dNMPs from nucleosides using the comparably expensive phosphate donor guanosine triphosphate (GTP). Due to a constant regeneration from the secondary phosphate donor acetyl phosphate only small amounts of GTP (2.5 mol%) were needed (Zou et al., 2013). Inorganic polyphosphate is the cheapest available secondary phosphate donor. However, energy-rich phosphate donors such as phosphoenolpyruvate (PEP) offer a significant thermodynamic advantage over other possible phosphate donors (Andexer and Richter, 2015). This allows ATP (re)generation in nearly quantitative fashion and prompted us to employ this system for this study.

**SCHEME 1:**
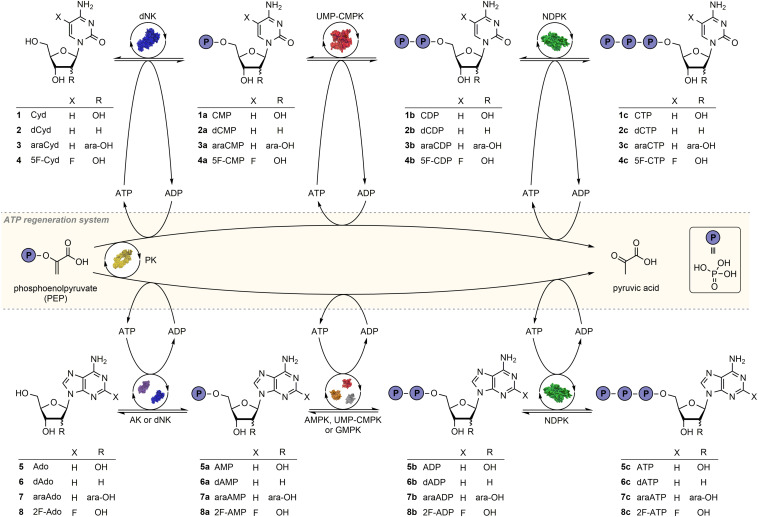
Reaction sequence for the phosphorylation of natural and modified nucleosides in a multi-enzyme cascade reaction with an ATP regeneration system based on pyruvate kinase and phosphoenolpyruvate. Depending on the substrate the enzymes *Drosophila melanogaster* deoxynucleoside kinase (dNK), adenosine kinase (AK), UMP-CMP kinase (UMP-CMPK), guanylate kinase (GMPK), adenylate kinase (AMPK), nucleoside diphosphate kinase (NDPK) and pyruvate kinase (PK) were applied. The protein structures shown belong to the PDB entries 2vqs (dNK), 3uq6 (AK), 2anc (GMPK), 1e4v (AMPK), 2cmk (UMP-CMPK), 2hur (NDPK) and 1pky (PK).

Inspired by the initial success of Hennig and colleagues, we herein report a pilot study to develop a generalizable method for the synthesis of natural and modified NTPs from nucleosides as cheaply available precursors (Scheme 1). Our modular one-pot four-enzyme cascade with a PEP-based phosphate donor recycling system allows the simple and efficient production of a wide range of NTPs, as demonstrated by examples bearing pyrimidine and purine bases as well as several different sugars. Nucleoside, NMP and NDP kinases were applied as biocatalysts using a standardized protocol. The application of different enzyme combinations allowed for a rapid adjustment to substrates of interest. Furthermore, the impact of a phosphate donor regeneration system on NTP yields was quantified for the first time. Significantly higher conversions were achieved for natural (4 to 9 times) and modified NTP products (4 to 6 times) using (d)ATP regeneration in a one-pot system, providing 4 NTPs in quantitative conversion. The present study lays the groundwork for future high-yielding syntheses of NTPs from nucleosides.

## Materials and Methods

### General Information

All chemicals and solvents were of analytical grade or higher and purchased, if not stated otherwise, from Sigma-Aldrich (Steinheim, Germany), Carl Roth (Karlsruhe, Germany), TCI Deutschland (Eschborn, Germany), Carbosynth (Berkshire, United Kingdom) or VWR (Darmstadt, Germany).

The following natural and modified nucleoside substrates were used: Natural pyrimidine and purine nucleoside substrates were cytidine (Cyd, **1**), deoxycytidine (dCyd, **2**), adenosine (Ado, **5**) and deoxyadenosine (dAdo, **6**). Sugar-modified nucleosides were arabinofuranosylcytosine (araCyd, **3**) known as the anti-leukemia drug Cytarabine and arabinofuranosyladenine (araAdo, **7**), a nucleoside antibiotic isolated from Streptomyces antibioticus. Base-modified nucleoside substrates were 5-fluorocytidine (5F-Cyd, **4**) and 2-fluoroadenosine (2F-Ado, **8**). Halogenated nucleotides like 5F-CTP (**4c**) are interesting building blocks for studies of secondary structures of DNA or RNA using NMR analysis (Hennig et al., 2007). 2F-Ado (**8**) is known as Fludarabine and its monophosphorylated form **8a** is used as an anti-cancer chemotherapy drug.

Wild-type nucleoside and nucleotide kinases were obtained from BioNukleo GmbH (Berlin, Germany) except for wide-spectrum deoxynucleoside kinase from *Drosophila melanogaster* (*Dm*dNK). The expression vector of *Dm*dNK was kindly provided by Prof. Birgitte Munch-Petersen (Roskilde University). According to the manufacturer the kinases possess the following substrate specificities: adenosine kinase (AK, NK14), guanylate kinase (GMPK, NMPK21) and adenylate kinase (AMPK, NMPK23) convert purine nucleoside/nucleotide substrates, while uridine monophosphate-cytidine monophosphate kinase (UMP-CMPK, NMPK22) and nucleoside diphosphate kinase (NDPK, NDPK32) accept both purine and pyrimidine nucleoside/nucleotide substrates. All enzymes obtained from BioNukleo were provided as stock solutions (0.1 to 1 mg/mL) and aliquots stored at −0.20°C until use. Pyruvate kinase (PK, P9136) was obtained from Sigma Aldrich as lyophilized powder, dissolved in 70 mM Tris–HCl pH 7.6 (1.74 mg/mL) and stored in aliquots at −0.20°C. All enzymes are active at 37°C and combinable in the same reaction buffer (70 mM Tris–HCl pH 7.6, 5 mM MgCl_2_).

### Expression of *Dm*dNK

Recombinant *Dm*dNK was expressed and purified as described before (Munch-Petersen et al., 2000). Briefly, the enzyme was produced as a GST-fusion protein using *Escherichia coli* BL21. Following glutathione sepharose purification the GST tag was cleaved of using thrombin and the enzyme was stored at −20°C with 8% glycerol, 1% Triton X-100 and 1 mM DTT.

### Enzymatic Cascade Reaction

Enzymatic cascade reactions were performed in a total volume of 50 μL with 70 mM Tris–HCl pH 7.6 (measured at 25°C), 5 mM MgCl_2_, 1 mM nucleoside substrate and 3.6 mM ATP. 2’-Deoxyadenosine triphosphate (dATP) was used as phosphate donor for reactions with adenosine as substrate to enable substrate and product differentiation. Reactions were started by adding 0.016-0.02 mg/mL (ratio 1:1:1; Supplementary Table SI) of each enzyme. Concentrations were chosen based on preliminary experiments. Reactions were incubated at 37°C in a thermocycler with a heatable lid. An equal volume of methanol (50 μL) was added after 19 h to stop the reaction. After centrifugation at 21.500 × *g* and 4°C for 15 min (Himac CT15RE, Hitachi, Tokyo, Japan) 75 μL of the quenched reaction mixture were diluted with 25 μL water and analyzed by high performance liquid chromatography (HPLC) as described below.

### Enzymatic Cascade Reaction With Phosphate Donor Recycling System

The phosphate donor regeneration system applied in this study consisted of a regeneration kinase (pyruvate kinase, PK) and a phosphate donor (phosphoenolpyruvate, PEP). Both ATP and dATP were accepted as substrates and were regenerated from the respective (deoxy)nucleoside diphosphates at 37°C. The enzymatic cascade reactions with phosphate donor recycling system were performed in a total volume of 50 μL with 70 mM Tris–HCl pH 7.6, 5 mM MgCl_2_, 1 mM nucleoside substrate, 3.6 mM ATP or dATP, 5 mM PEP and 0.17 mg/mL PK. Reactions were started by adding 0.016-0.02 mg/mL of each of the enzymes (ratio 1:1:1, Supplementary Table SI) and were incubated at 37°C for 19 h. The reactions were stopped by adding equal volumes of methanol (50 μL) followed by centrifugation at 21.500 × *g* and 4°C for 15 min. After centrifugation, 75 μL of the samples were mixed with 25 μL water and analyzed by HPLC.

### Time Course of the Enzymatic Cascade Reaction With and Without Regeneration System

To analyze the enzymatic reactions over time, reaction volumes were scaled up to 0.7 mL with and 1 mL without ATP regeneration system. Enzymatic reactions were performed as described above, but incubated in a thermoblock without lid-heating. The reaction tubes were incubated at 300 rpm for 33 h. Regular samples of 50 μL were taken and mixed with equal volumes of methanol followed by centrifugation at 21.500 × *g* and 4°C for 15 min. After centrifugation, 75 μL of the samples were mixed with 25 μL water and analyzed by HPLC.

### High Pressure Liquid Chromatography (HPLC)

Samples from nucleoside/nucleotide kinase reactions were analyzed by HPLC-DAD (Agilent 1200 series) with a detection wavelength of 260 nm using a Kinetex Evo column (C18, 100 A, 250 × 4.6 mm, Phenomenex, Aschaffenburg, Germany). The method was adapted from literature (Ryll and Wagner, 1991). The flow rate was set to 1 mL/min at 34°C and the gradient consisted of A (0.1 M KH_2_PO_4_/K_2_HPO_4_, 8 mM tetrabutylammonium bisulfate, pH ca. 5.4) and B (70% A, 30% methanol): 0 min – 80% A, 4 min – 80% A, 14 min – 40% A, 26 min – 38% A, 26.5 min – 80% A, and 29 min – 80% A. Reactions with compound 2F-Ado (**8**) as substrate were analyzed using a prolonged gradient: 0 min – 80% A, 4 min – 80% A, 14 min – 40% A, 35 min – 36.5% A, 35.5 min – 80% A, and 38 min – 80% A. Natural nucleosides and nucleotides as well as nucleoside analogs were identified using authentic standards. Nucleotide analog peaks were assigned based on analogy of retention times and characteristic UV absorption spectra. Typical retention times [min] were: Cyd (**1**) – 2.8, CMP (**1a**) – 3.3, CDP (**1b**) – 5.5, CTP (**1c**) – 12.9, dCyd (**2**) – 2.6, dCMP (**2a**) – 3.4, dCDP (**2b**) – 6.3, dCTP (**2c**) – 14.5, araCyd (**3**) – 3, araCMP (**3a**) – 3.5, araCDP (**3b**) – 6.2, araCTP (**3c**) – 13.7, 5F-Cyd (**4**) – 2.8, 5F-CMP (**4a**) – 3.2, 5F-CDP (**4b**) – 5.4, 5F-CTP (**4c**) – 12.8, Ado (**5**) – 5.9, AMP (**5a**) – 7.3, ADP (**5b**) – 14, ATP (**5c**) – 20.8, dAdo (**6**) – 6.4, dAMP (**6a**) – 10.1, dADP (**6b**) – 17.3, dATP (**6c**) – 26.3, araAdo (**7**) – 5.5, araAMP (**7a**) - 8, araADP(**7b**) – 15.5, araATP (**7c**) – 23, 2F-Ado (**8**) - 9, 2F-AMP (**8a**) – 12.8, 2F-ADP (**8b**) – 19.3, 2F-ATP (**8c**) – 29.9 (Supplementary Figures 2, 3).

Conversion was calculated as:

(1) $$Conversion\left(X\right)\left[\%\right]=100\times \frac{P_{X}}{P_{t\,o\,t\,a\,l}}$$

where *P*_*X*_ is the peak area of compound X and *P*_*total*_ is the sum of all peak areas of the substrate and product(s) in the reaction. Consumption of the cofactor (d)ATP was not considered in the calculation.

## Results

### Time Course of Enzymatic Cascade Reactions Using Nucleoside and Nucleotide Kinases

We aimed to use a modular enzymatic cascade system to synthesize a range of natural as well as sugar- and base-modified NTPs using the respective nucleosides as substrates. Specifically, starting from nucleosides **1**-**8** we aimed to access cytidine-5′-triphosphate (**1c**) and its 2’-deoxy (**2c**), arabinofuranosyl (**3c**) and 5-fluoro analogs (**4c**) as well as adenosine-5′-triphosphate (**5c**) and its 2′-deoxy (**6c**), arabinofuranosyl (**7c**) and 2-fluoro analogs (**8c**) through a one-pot enzyme cascade reaction (Scheme 1). Based on literature data (Serra et al., 2014) we anticipated that a slight excess of ATP would enable access to the NTP products and performed initial experiments with 1.2 equivalents of ATP per product phosphate (3.6 eq. per nucleoside).

**FIGURE 1:**
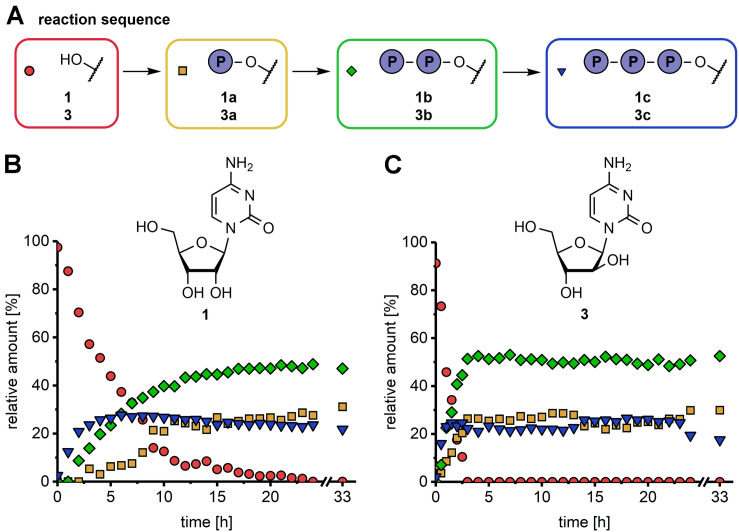
Time course for the synthesis of natural (**B**, 1a–1c) and modified nucleotides (**C**, 3a–3c) in an one-pot multi-enzyme cascade reaction **(A)**. Experimental conditions: 1 mM substrate, 3.6 mM phosphate donor, 70 mM Tris–HCl pH 7.6, 5 mM MgCl_2_, 0.016-0.02 mg/mL of each enzyme, 37°C. The formation of NMP (yellow square), NDP (green diamond) and NTP (blue triangle) was analyzed over 33 h using the natural nucleoside cytidine (**1**, **B**) and sugar-modified nucleoside analog arabinosylcytosine (**3**, **C**) as substrates (red circle). The enzyme combination *Dm*dNK/UMP-CMPK/NDPK was applied. The first datapoint was taken 30 s after reaction initiation.

In a first set of experiments we sought to confirm if the envisioned enzyme cascade could deliver the expected NTP products and to identify suitable reaction times for the synthesis of these nucleotides. To this end the time course of the conversions of cytidine (Cyd, **1**) and arabinosyl cytosine (araCyd, **3**) (Figure 1A) were analyzed over a period of 33 h as exemplary transformations. In both reactions the enzymes *Dm*dNK, UMP-CMPK and NDPK were used as catalysts with ATP serving as the phosphate donor. Enzymes were either chosen based on substrate specificities given by the supplier (UMP-CMPK, NDPK) or due to a known wide substrate spectrum (*Dm*dNK) (Serra et al., 2014).

HPLC analysis of the reaction samples revealed the formation of the desired products, confirming the successful implementation of the enzymatic cascade. With the applied enzyme combination nucleoside analog **3** was faster converted than the natural counterpart **1**. In both reactions NTP products were already detectable at the first time point of sampling (1 h for **1** and 0.5 h for **3**) and maximum concentration was reached after 2 h (25% araCTP, **3c**) and 6 h (28% CTP, **1c**) (Figures 1B,C). Although the amount of the NTP products **1c** and **3c** did not change further at that point, conversion of the nucleosides to the NDPs **1b** and **3b** continued until 4 and 19 h after reaction initiation. An equilibrium of the reaction was reached after 19 and 4 h for **1** and **3**, respectively, confirmed by datapoints after 24 and 33 h showing no change in reactant concentrations. At the end of the reaction at 33 h, the final nucleotide ratios (nucleoside:NMP:NDP:NTP) were 0:31:47:22% for **1** and 0:30:53:18% for **3**, respectively. These experiments showed that the selected enzymes indeed delivered the desired phosphorylated products directly from the nucleoside through cascade catalysis. Thus, stability and performance of these biocatalysis under the selected conditions encouraged further exploration of this system. Furthermore, since the equilibrium was reached after 19 h even for the rather sluggish reaction of these enzymes with **1**, we selected this time for the following reactions.

### One-Pot Enzymatic Cascade Reaction to Produce Natural and Modified Nucleoside Triphosphates

**FIGURE 2:**
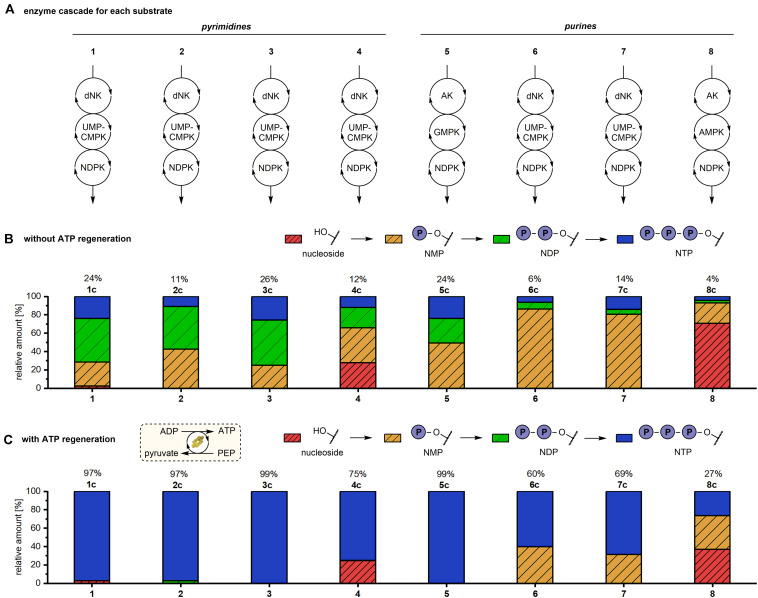
Production of natural and modified NTPs in an one-pot multi-enzyme cascade reaction with and without phosphate donor regeneration system. Experimental conditions: 1 mM substrate, 3.6 mM phosphate donor, 70 mM Tris–HCl pH 7.6, 5 mM MgCl_2_, 0.016-0.02 mg/mL each enzyme, 37°C, 19 h. Reactions with phosphate donor regeneration **(C)** additionally contained 5 mM phosphoenolpyruvate and 0.17 mg/ml pyruvate kinase. **(A)** Eight natural and modified nucleosides were used as substrates for the multi-enzyme cascade reactions. Applied enzymes: dNK = *Drosophila melanogaster* deoxynucleoside kinase, AK = adenosine kinase, UMP-CMPK = UMP-CMP kinase, GMPK = guanylate kinase, AMPK = adenylate kinase, NDPK = nucleoside diphosphate kinase. **(B,C)** Percentages of Nucleoside, NMP, NDP and NTP without **(B)** and with **(C)** phosphate donor regeneration system. Without ATP regeneration system dCTP conversion could not be determined by HPLC and was therefore calculated based on the dCMP and dCDP conversions.

To investigate if this one-pot cascade system is a generalizable method for the synthesis of natural and modified NTPs a wider spectrum of substrates was tested (Scheme 1 and Figure 2A). Therefore, base- and sugar-modified derivatives of both cytidine (**2**-**4**) and adenosine (**6**-**8**) were subjected to enzymatic phosphorylation to access the respective NTPs in one pot. ATP was used as phosphate donor, except for reactions with adenosine as substrate, where dATP was applied as phosphate donor to enable differentiation between substrates and products. All reactions were run for 19 h at 37°C before analysis by HPLC. In initial experiments to produce pyrimidine NTPs **1c**-**4c** *Dm*dNK, UMP-CMPK and NDPK were used as biocatalysts while reactions toward purine nucleotides **5c**-**8c** reactions were performed using AK, GMPK, and NDPK.

Reactions with pyrimidine substrates yielded 11 to 26% of the respective NTP (Figure 2B). In reactions with **1**-**3** nearly no nucleoside substrate was left after 19 h reaction time. In contrast, 28% residual **4** was detected at the end of the reaction suggesting a lower activity of first cascade enzyme *Dm*dNK toward the base-modified cytidine analog. In reactions with **1** and **3** comparable amounts of NTP (24 to 26%), NDP (46 to 49%) and NMP (25 to 26%) were formed. With **2** as the substrate only 11% of the corresponding triphosphate **2c** were formed. Conversions to the NMP **2a** and the NDP **2b** of 43% and 46% were observed, respectively. These observations suggest that the second cascade enzyme UMP-CMPK and/or the final enzyme NDPK prefer ribo- and arabino-sugar moieties over deoxyribose.

Contrary to the initial success of the reactions with pyrimidines **1**-**4**, we failed to detect a conversion of purine nucleosides **6**-**8** with the applied enzyme system (Table 1). Only Ado (**5**) was efficiently transformed to the corresponding NTP **5c** in 24% conversion.

**TABLE 1:** Phosphorylation of adenosine derivatives in a one-pot kinase cascade reaction using different enzyme combinations.

Substrate	Kinase 1		Kinase 2			Kinase 3	Relative amount [%]*			
				
	*Dm*dNK	AK	GMPK	UMP-CMPK	AMPK	NDPK	Nucleoside	NMP	NDP	NTP
Ado (**5**)		**x**	**x**			**x**	**0**	**49**	**27**	**24**
dAdo (**6**)		x	x			x	100	0	0	0
	x		x			x	0	93	4	3
	**x**			**x**		**x**	**0**	**86**	**8**	**6**
araAdo (**7**)		x	x			x	100	0	0	0
	x		x			x	0	100	0	0
	**x**			**x**		**x**	**0**	**80**	**5**	**14**
2F-Ado (**8**)		x	x			x	96	4	0	0
	x		x			x	76	24	0	0
	x			x		x	77	23	0	0
		**x**			**x**	**x**	**71**	**22**	**3**	**5**

*Experimental conditions: 1 mM substrate, 3.6 mM phosphate donor, 70 mM Tris HCl pH 7.6, 5 mM MgCl_2_, 0.016-0.02 mg/mL of each enzyme, 37°C, 19 h. The enzyme combinations resulting in the largest NTP formation are depicted in bold. Applied enzymes: *Dm*dNK = *Drosophila melanogaster* deoxynucleoside kinase, AK = adenosine kinase, UMP-CMPK = UMP-CMP kinase, GMPK = guanylate kinase, AMPK = adenylate kinase, NDPK = nucleoside diphosphate kinase.

Therefore, we aimed to improve the product yields for these compounds by employing different enzyme combinations. The exchange of the first cascade enzyme AK with the more promiscuous *Dm*dNK resulted in over 90% conversion of the 2’-deoxyribosyl nucleoside **6** and the arabinosyl nucleoside **7** to the corresponding NMP (Table 1). Additionally, the formation of the fluorinated species **8** to the NMP **8a** was improved with this strategy, increasing the conversion from 4 to 22% (Table 1). However, the near absence of NDP products in all reactions indicated that conversion from the respective NMP was a limiting factor. Therefore, the second cascade enzyme GMPK was replaced with the more promiscuous UMP-CMPK. This substitution enabled smooth conversion of NMPs **6a** and **7a** to the respective NDPs and NTPs, yielding **6c** and **7c** in 6% and 14% conversion, respectively. Nonetheless, UMP-CMPK showed no activity on the fluorinated **8a** in the one-pot cascade reaction, which delivered only the monophosphorylated product and no detectable formation of the NDP and NTP. Lastly, another purine-specific nucleotide kinase, namely AMPK, when incorporated in the cascade reaction yielded 5% conversion of the fluorinated purine nucleoside to its triphosphate **8c** (Table 1).

Taken together, these experiments demonstrate the general feasibility to access several NTPs from their corresponding nucleosides in a one-pot cascade reaction by adjusting the applied enzymes. However, the conversion to the NTPs **1c**-**8c** did not reach satisfactory levels (≤26% in all cases). This spurred us to investigate if a PEP-based (d)ATP recycling system could be employed to improve conversions.

### One-Pot Enzymatic Cascade Reaction to Produce Natural and Modified Nucleoside Triphosphates Using a Phosphate Donor Recycling System

Next, the reactions described so far were repeated under the same conditions with a (d)ATP recycling system based on PK and PEP to investigate the impact of cofactor recycling on the one-pot enzymatic cascade reactions. A ratio of substrate to PEP to (d)ATP of 1:5:3.6 was applied, with the aim to avoid the formation of (d)ADP. In experiments with and without (d)ATP regeneration the same substrate to (d)ATP ratio was used to enable direct a comparison of the substrate conversion in both reaction set ups. In literature it was shown before that different ATP to substrate ratios influenced product formation (Serra et al., 2014). All reactions were run for 19 h with 5 equivalents of PEP per nucleoside.

The incorporation of the coupled enzymatic (d)ATP recycling reaction had a strikingly positive effect on the NTP yield for all tested substrates, as determined by HPLC (Figure 2C). Natural nucleosides **1**, **2**, **3** and **5** were nearly completely converted to the respective (d)NTPs (≥97%), which is four to nine times higher than the conversions observed without (d)ATP recycling (6 to 24%).

Pyrimidine nucleoside analogs **3** and **4** were converted to their corresponding NTPs in >99% and 75%, respectively. Non-etheless, 25% residual **4** was detected in the reaction mixture after 19 h reaction time. Without ATP recycling approximately the same amount of residual nucleoside substrate was detected while only 12% NTP were formed (Figure 2B), indicating that conversion of **4** to the monophosphate **4a** clearly represents a bottleneck in the cascade.

HPLC analysis further revealed that the NTP yield of modified purine nucleotides **7c** and **8c** was improved by factors of 5 and 6, respectively. The conversion to **7c** was increased from 14% to 69% (Figures 2B,C). By including the ATP recycling system, the conversion to **8c** increased from 4 to 27% while the percentage of the remaining nucleoside substrate **8** decreased from 71 to 37%. This observation highlights that the inadequate conversion of these nucleosides without cofactor recycling was not due to a kinetically limited step (as seen for **4**), but due to thermodynamic limitations.

The incorporation of the phosphate donor regeneration system further led to reduced reaction times. The time courses of conversion of **1** and **3** to **1c** and **3c** with the ATP regeneration system showed a 4 to 6 times faster product formation compared to our initial experiments (Supplementary Figure S1). The maximum amount of both NTPs was approximately 25% without ATP regeneration after 6 and 2 h. HPLC analysis indicated that those NTP yields were already reached after 1 h (**1c**) and 30 min (**3c**) with ATP regeneration. NTP formation continued after that and reached a maximum of >97% after 18 h (**1c**) and 4 h (**3c**) when including the ATP regeneration system.

## Discussion

The one-pot enzyme cascade reaction described in this study allows for the efficient synthesis of natural and modified NTPs from nucleosides as substrates. A standardized protocol was used which includes a fixed reaction buffer, enzyme amount and reaction time. This system succeeded in delivering all desired NTPs in various conversions up to 26%. The integration of a phosphate donor regeneration system further improved product yields and reduced reaction times. Highest NTP formation was reached with this system using nucleosides **1**, **2**, **3** and **5** (all >97%).

The synthesis of NTPs from nucleosides offers the advantage that nucleosides are readily available and inexpensive substrates. Furthermore, the use of purified enzymes promises minimization of side reactions and an easier purification process. The feasibility of this approach for the synthesis of modified NTPs has already been successfully demonstrated, albeit only for selected examples. For example, Hennig et al. synthesized 5F-CTP (**4c**) in a one-pot approach with near complete conversion (Hennig et al., 2007). We were also able to show that high conversions can be achieved with nucleoside **4**, since the corresponding triphosphate **4c** was produced with 75% yield after integration of an ATP regeneration system. Nonetheless, a modular enzyme system for the synthesis of multiple NTPs has not been reported to date. We addressed this gap by assaying a number of kinases for their potential to furnish NTPs directly from nucleosides through cascade catalysis.

A crucial factor for the development of a generalizable method is the identification of suitable enzymes. This became evident in the synthesis of ATP derivatives in this study. Significantly higher product yields were observed with *Dm*dNK and UMP-CMPK compared to AK and GMPK as the latter were only suitable enzymes for the phosphorylation of adenosine (**5**). This observation is in good accordance with the available literature, showing that AK and GMPK performed suboptimally with deoxyribose- and arabinose-derivatives (Van Rompay et al., 2000, 2003), while *Dm*dNK and UMP-CMPK were described to accept a wide variety of sugar-modified substrates (Liou et al., 2002; Serra et al., 2014). Interestingly, the time course experiments performed with Cyd (**1**) and araCyd (**3**) even revealed a higher reaction rate of the applied enzymes (*Dm*dNK, UMP-CMPK, NDPK) toward the sugar-modified pyrimidine nucleoside analog **3** compared to the natural counterpart **1**. We ascribed this to *Dm*dNK’s preference for deoxyribose- and arabinose-sugar moieties over ribose-containing nucleosides (Munch-Petersen et al., 2000) as well as the fact that CMP (**1a**) but not araCMP (**3a**) leads to significant substrate inhibition of UMP-CMPK (Pasti et al., 2003). In summary, wide-spectrum nucleoside kinases proved most suitable for the synthesis of sugar-modified nucleotides.

In contrast, for the synthesis of base-modified nucleotides like **4c** the use of a pyrimidine specific nucleoside kinase as the first cascade enzyme might be advantageous over the wide substrate-spectrum *Dm*dNK. Mammalian enzymes like uridine-cytidine kinase (UCK) and deoxycytidine kinase (dCK) were described to phosphorylate a variety of base-modified nucleoside analogs like **4**, 5-fluororuridine, 2’-deoxy-5-methylcytidine and 2-thiocytidine (Van Rompay et al., 2003; Hazra et al., 2010). Thus, UCK might be superior over dCK and the *Dm*dNK employed in this study for the phosphorylation of **4** because of a higher activity of UCK toward ribonucleoside substrates (Van Rompay et al., 2003). To identify suitable enzymes for the application in one-pot cascade reactions automated high-throughput assays might be a suitable tool. As shown for nucleoside kinases high-throughput assays allow for the fast and accurate activity screening using a large number of enzymes (Hellendahl/Fehlau et al., in preparation) and we expect future improvements in this area that enable efficient phosphorylation of previously challenging substrates.

Another approach to overcome limitations in the phosphorylation of base-modified nucleosides is to start syntheses from natural sugars. Nucleotide **8c** was produced in near quantitative yield applying ribokinase and phosphoribosyl pyrophosphate (PRPP) synthetase for sugar activation followed by adenine phosphoribosyl transferase, adenylate kinase and creatine kinase (Scott et al., 2004). Others have followed a similar approach to produce 8-azaguanosine triphosphate and 5-fluorouridine triphosphate with high yields (Da Costa et al., 2007; Hennig et al., 2007). Although these enzymes show good activity toward base-modified substrates, they, however, are not applicable for cytosine derivatives (Scheit and Linke, 1982; Hennig et al., 2007) and show limited usefulness for the preparation of sugar modified NTPs (Esipov et al., 2016).

Cofactor recycling systems are commonly used in industrial biotransformation processes where costly cofactors like NAD/H would lead to uneconomic production routes (Weckbecker et al., 2010). As an additional benefit reaction equilibria are shifted to favor product formation through cofactor (re)generation and the reduction of product inhibition (Abu and Woodley, 2015). Although (d)ATP recycling systems have been applied for the synthesis of natural and modified nucleotides a comparison to reactions without these has never been shown (Hennig et al., 2007; Zou et al., 2013) and the benefit of these strategies has remained elusive. In this study, a strong beneficial effect was demonstrated for all tested compounds. NTP product yields increased by a factor of 4 (for **3c**) to 9 (for **6c**) using a PEP-based phosphate donor regeneration system. This observation provides clear evidence for the usefulness of employing ATP recycling systems for NTP synthesis since half of the nucleosides in our study were quantitatively converted to the respective triphosphate.

It remains to be investigated to which extent the applied regeneration enzyme takes part in the last cascade step converting NDPs and NTPs. Pyruvate kinase, acetate kinase and polyphosphate kinase possess wide substrate spectra (Ishige et al., 2001; Gao et al., 2008; Zou et al., 2013) and may be envisioned to serve a twofold purpose. In addition to regenerating the phosphate donor, PK might replace the NDPK in some reactions. For example, the parallel use of a pyruvate kinase for NTP formation and phosphate donor regeneration was demonstrated by Hennig and coworkers in the cascade synthesis of 5F-UTP and -CTP (Hennig et al., 2007). Furthermore, in order to achieve economic (d)ATP recycling in the established cascade system future studies could focus on optimizing the ratio of phosphate donor to substrate by applying substoichiometric (d)ATP concentrations as has been successfully shown before for selected single and multi-enzyme syntheses (Hennig et al., 2007; Zou et al., 2013).

## Conclusion

We established a modular enzymatic cascade for the synthesis of NTPs from nucleosides in one pot. With (d)ATP as the phosphate donor all desired NTPs were accessed in low yield. The application of a (d)ATP regeneration system resulted in a shift of the reaction equilibrium toward the desired product NTP. Our approach, for the first time, allows for the efficient production of both natural and modified NTPs in high conversion with a standardized protocol after identifying a suitable enzyme combination. Thus, the present study lays the foundation for future high-yielding biocatalytic syntheses of NTPs from nucleosides.

## Data Availability Statement

The raw data supporting the conclusions of this article will be made available by the authors, without undue reservation.

## Author Contributions

MF performed experiments and data analysis and wrote the manuscript. FK revised and illustrated the manuscript together with AW. KH and JS assisted with experiments. PN and AW coordinated the project and were involved in study design, data analysis, and revision and submission of the manuscript. All authors contributed to the article and approved the submitted version.

## Conflict of Interest

BioNukleo GmbH is a spin-out of the Chair of Bioprocess Engineering at the TU Berlin with AW as CEO, FK and JS as employees, and MF and PN as board members. The remaining author declares that the research was conducted in the absence of any commercial or financial relationships that could be construed as a potential conflict of interest.
