# Corrigendum: Modular Enzymatic Cascade Synthesis of Nucleotides Using a (d)ATP Regeneration System

**DOI:** 10.3389/fbioe.2020.606584

**Published:** 2020-10-26

**Authors:** Maryke Fehlau, Felix Kaspar, Katja F. Hellendahl, Julia Schollmeyer, Peter Neubauer, Anke Wagner

**Affiliations:** ^1^Chair of Bioprocess Engineering, Institute of Biotechnology, Faculty III Process Sciences, Technische Universität Berlin, Berlin, Germany; ^2^BioNukleo GmbH, Berlin, Germany

**Keywords:** enzymatic cascade synthesis, nucleoside-5′-triphosphate, one-pot multi-enzyme reaction, nucleotide analog, nucleotide kinase, nucleoside kinase, modular, ATP regeneration system

In the original article, there was an error. We did not receive the expression plasmid of *Dm*dNK from Daniela Ubiali (which is written 2x in the manuscript), but from Prof. Munch-Petersen.

A correction has been made to **Materials and Methods, General Information**, paragraph 3.

The corrected paragraph appears below:

“Wild-type nucleoside and nucleotide kinases were obtained from BioNukleo GmbH (Berlin, Germany) except for wide-spectrum deoxynucleoside kinase from *Drosophila melanogaster* (*Dm*dNK). The expression vector of *Dm*dNK was kindly provided by Prof. Birgitte Munch-Petersen (Roskilde University). According to the manufacturer the kinases possess the following substrate specificities: adenosine kinase (AK, NK14), guanylate kinase (GMPK, NMPK21) and adenylate kinase (AMPK, NMPK23) convert purine nucleoside/nucleotide substrates, while uridine monophosphate-cytidine monophosphate kinase (UMP-CMPK, NMPK22) and nucleoside diphosphate kinase (NDPK, NDPK32) accept both purine and pyrimidine nucleoside/nucleotide substrates. All enzymes obtained from BioNukleo were provided as stock solutions (0.1 to 1 mg/mL) and aliquots stored at −20°C until use. Pyruvate kinase (PK, P9136) was obtained from Sigma Aldrich as lyophilized powder, dissolved in 70 mM Tris–HCl pH 7.6 (1.74 mg/mL) and stored in aliquots at −20°C. All enzymes are active at 37°C and combinable in the same reaction buffer (70 mM Tris–HCl pH 7.6, 5 mM MgCl_2_).”

A correction has been made to **Acknowledgments**.

The corrected paragraph appears below:

“We thank Prof. Munch-Petersen for supplying *Drosophila melanogaster* deoxynucleoside kinase plasmid (*Dm*dNK). We thank the Open Access Publishing funds of TU Berlin for the support of this publication.”

The authors apologize for this error and state that this does not change the scientific conclusions of the article in any way. The original article has been updated.

