# Supplementary material for: Modular Enzymatic Cascade Synthesis of Nucleotides Using a (d)ATP Regeneration System
Source: Front Bioeng Biotechnol. 2020 Aug 6;8:854. doi: 10.3389/fbioe.2020.00854 (PMC7438870; doi:10.3389/fbioe.2020.00854)
Supplement: Supplementary file 1 [file Image_1.pdf]

Supplementary Material

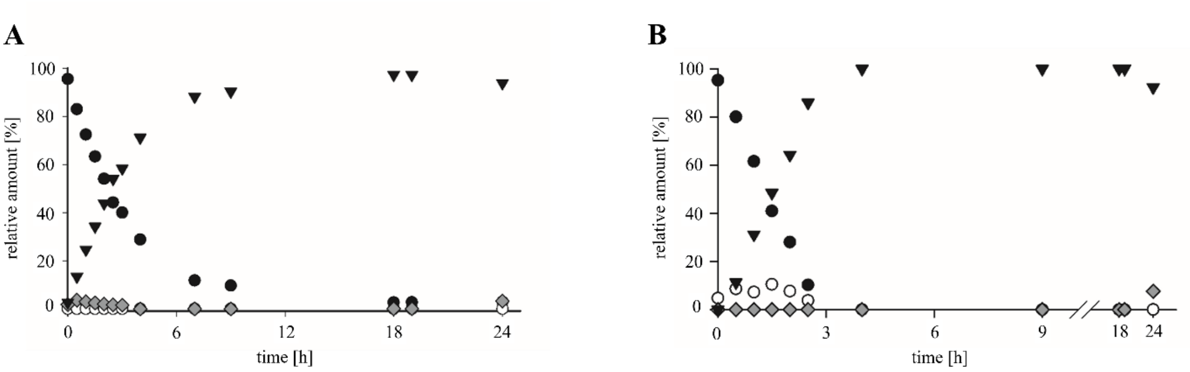

Supplementary Figure 1. Time course for the synthesis of natural (**A**, **1a-c**) and modified nucleotides (**B**, **3a-c**) in an one-pot multi-enzyme cascade reaction. The formation of NMP ○, NDP ◆ and NTP ▼was analyzed over 24 h using the natural nucleoside cytidine (**1**, **A**) and sugar-modified nucleoside analog arabinosylcytosine (**3**, **B**) as substrates (●). The enzyme combination *Dm*dNK / UMP-CMPK / NDPK was applied. The first datapoint was taken 30 s after reaction initiation. Experimental conditions: 1 mM substrate, 3.6 mM phosphate donor, 70 mM Tris HCl pH 7.6, 5 mM $MgCl_2$, 0.016−0.02 mg/mL each cascade enzyme, 5 mM phosphoenolpyruvate and 0.17 mg/mL pyruvate kinase, 37°C.

Table SI. Enzymes and enzyme concentrations applied for the synthesis of natural and modified NTPs in the absence and presence of a (d)ATP recycling system.

**Compound**	**NK**		**NMPK**		**NDPK**	
	Enzyme	Conc. [mg/ml]	Enzyme	Conc. [mg/ml]	Enzyme	Conc. [mg/ml]
**1c**	*Dm*dNK	0.016	UMP-CMPK	0.016	NDPK	0.016
**2c**	*Dm*dNK	0.016	UMP-CMPK	0.016	NDPK	0.016
**3c**	*Dm*dNK	0.016	UMP-CMPK	0.016	NDPK	0.016
**4c**	*Dm*dNK	0.020	UMP-CMPK	0.020	NDPK	0.020
**5c**	AK	0.020	GMPK	0.020	NDPK	0.020
**6c**	*Dm*dNK	0.016	UMP-CMPK	0.016	NDPK	0.016
**7c**	*Dm*dNK	0.016	UMP-CMPK	0.016	NDPK	0.016
**8c**	AK	0.020	AMPK	0.020	NDPK	0.020

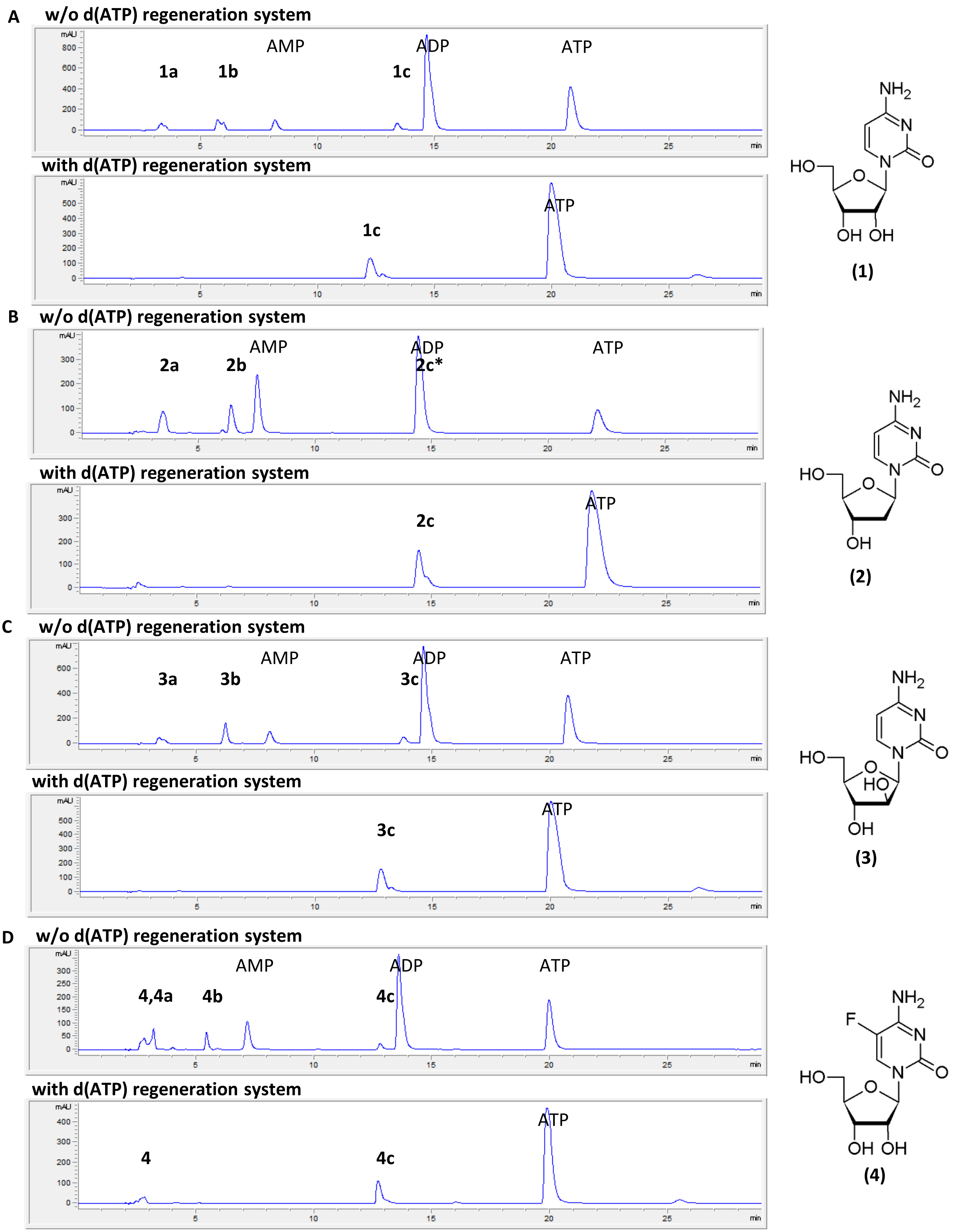

Supplementary Figure 2. HPLC chromatograms obtained during the synthesis of natural and modified pyrimidine NTPs 1c (A), 2c (B), 3c (C), 4c (D) in the absence and presence of the (d)ATP regeneration system.

) Compound **2 top figure (without ATP regeneration): The dCTP (2c) peak overlays with ADP peak and therefore cannot be evaluated. Therefore, the dCTP amount was calculated from dCMP and dCDP amounts. With ATP regeneration (lower figure) there was no ADP present judged by the UV absorption spectrum of the respective peak 2c.*

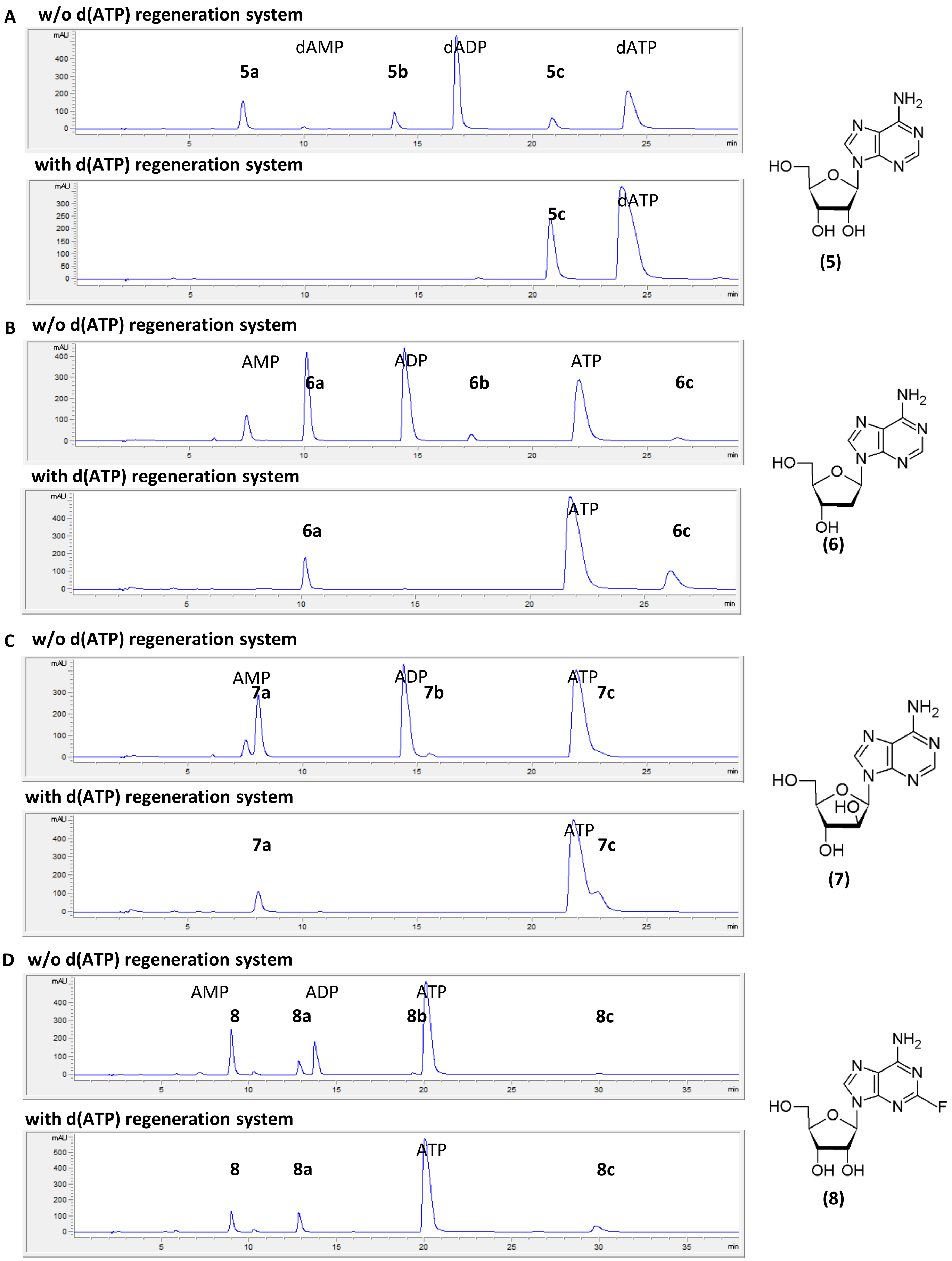

Supplementary Figure 3. HPLC chromatograms obtained during the synthesis of natural and modified purine NTPs 5c (A), 6c (B), 7c (C), 8c (D) in the absence and presence of the (d)ATP regeneration system.
